# von Hippel-Lindau Syndrome and Secondary Hypertension: A Case Report

**DOI:** 10.7759/cureus.61702

**Published:** 2024-06-05

**Authors:** Sharan Bose, Kandasamy Venkataraju Rajalakshmi, Ananthakumar Perumal Kumaresan, Jibin Simon

**Affiliations:** 1 Internal Medicine, Saveetha Institute of Medical and Technical Sciences, Saveetha University, Chennai, IND

**Keywords:** secondary hypertension, retinoblastoma, severe hypertension, adrenal pheochromocytoma, von hippel-lindau disease (vhl)

## Abstract

von Hippel-Lindau (VHL) syndrome (OMIM #193300) is an autosomal dominant disorder with incomplete penetrance occurring due to a mutation in the *VHL* gene present on chromosome 3. We present the case of a 21-year-old male with a history of retinoblastoma presenting with intermittent headaches for one month. He was a known hypertensive and his blood pressure on presentation was 180/100 mmHg. A secondary cause for his hypertension was sought. Multiple cysts in his pancreas, both his kidneys, and a mass in the right suprarenal fossa were detected on an abdominal ultrasonogram and a subsequent computed tomography scan of the abdomen. VHL and a pheochromocytoma were suspected, and a positron emission tomography-computed tomography scan was done which collaborated with the above findings. The presence of multiple cystic lesions in the pancreas and kidneys, especially in an individual with a family history of VHL syndrome, should alert the physician to the possibility of VHL syndrome. The need for evaluation of causes for hypertension, especially in young individuals with resistant hypertension, is also highlighted.

## Introduction

The von Hippel-Lindau (VHL) syndrome is an inherited, autosomal dominant syndrome characterized by several benign and malignant tumors. It occurs due to a defect in the *VHL* tumor suppressor gene present on chromosome 3p [1]. The incidence is roughly 1 in 36,000 individuals [2]. It is characterized by hemangioblastomas of the retina, spinal cord, and brain; renal cysts and clear cell carcinoma of the kidneys; pheochromocytomas or paragangliomas; pancreatic cysts; and endolymphatic sac tumors of the middle ear, among others [3]. The manifestations and clinical presentations are highly variable even within members of the same family, even among those sharing the same pathogenic variant.

Patients with VHL present with varying symptoms in different age groups, ranging from pediatric to early adulthood age groups, based on the type and extent of tumors. It is diagnosed based on clinical suspicion, and confirmation is commonly done by molecular testing and imaging techniques. About 50-60% of patients experience symptoms by the age of 25 years. The incidence of pheochromocytoma is only around 10-20% among patients with VHL [4]. These pheochromocytomas may be adrenal or extra-adrenal. This case report describes a man in his 20s, with a history of a retinoblastoma presenting to us with resistant hypertension.

## Case presentation

A 21-year-old male came to us with a one-month history of holocranial headaches and uncontrolled hypertension despite treatment with an angiotensin receptor blocker and a calcium channel blocker. Noteworthy past medical history included left eyeball evisceration for a retinoblastoma in 2006. No further workup was performed at the time. There was no family history of hypertension or other malignancies. He was born of a second-degree consanguineous marriage. On examination, he was conscious, oriented, and afebrile. He was moderately built and nourished. His blood pressure was 180/100mmHg and his pulse rate was 96/minute. The abdomen was soft, and no tenderness or organomegaly was detected. Bowel sounds were heard normally. His nervous system examination was normal, revealing no focal neurological deficits. Other systemic examinations were unremarkable.

Basic laboratory assessments revealed leukocytosis and unremarkable liver function tests, aligning with standard reference ranges. Given the early onset and severity of hypertension, an extensive investigative journey ensued. His thyroid function tests were within normal limits. Other relevant investigations for the hypertension workup are shown in Table 1.

**Table 1:** Summary of investigations done for the secondary hypertension workup. CA 19-9: carbohydrate antigen 19-9

Parameter	Value	Reference range
8 am cortisol	7.57 µg/dL	6.2–19.4 µg/dL
Serum aldosterone	187.7 pg/mL	25–315 pg/mL
Plasma metanephrines	112.4 pg/mL	<65 pg/mL
Plasma normetanephrines	624.5 pg/mL	<196 pg/mL
Urinary vanillyl mandelic acid	7.2 mg/g	2–7 mg/g
CA 19-9	22.3 U/mL	<37 U/mL

**Figure 1:**
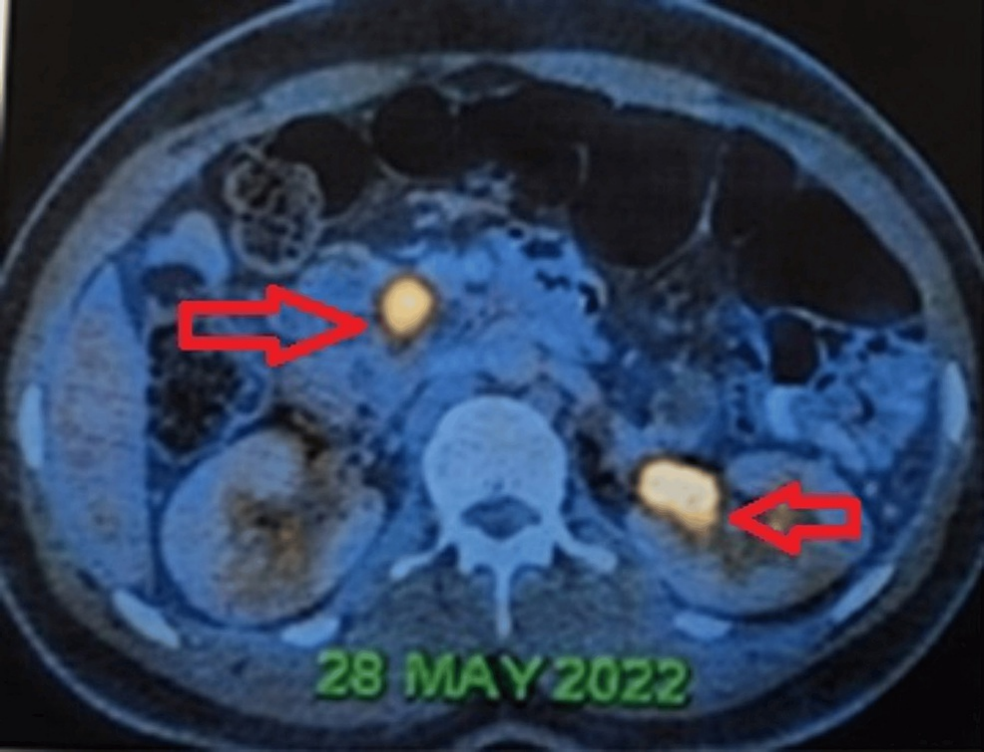
Positron emission tomography-computed tomography image showing cystic lesions in the pancreas, with lesions having increased metabolic activity in the head/uncinate process of the pancreas and interpolar region of the left kidney.

**Figure 2:**
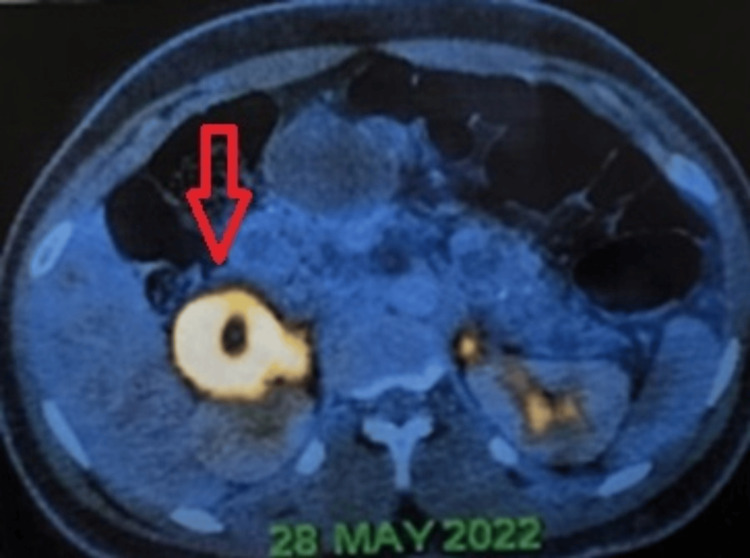
Positron emission tomography-computed tomography image showing a lesion with increased metabolic activity in the right suprarenal fossa.

Ultrasonography and contrast-enhanced computed tomography (CT) of the abdomen delineated multiple cystic lesions in the pancreas, neoplastic lesions in the right adrenal and prevertebral regions, and Bosniak type 4 cystic lesions in the kidney. Positron emission tomography-computed tomography (Figures 1, 2) corroborated metabolically active lesions in these areas. Hormonal profiling demonstrated elevated plasma metanephrines and normetanephrines, indicative of adrenal involvement. His CT of the brain was normal.

He was started on alpha-blockers followed by beta-blockers and his hypertension was brought under control. A right adrenalectomy was done to remove the mass in the adrenal gland. Antihypertensives were given before surgery and continued during the procedure as well. The surgery and postoperative periods were uneventful. He is now under regular follow-up and is symptomatically better.

## Discussion

The VHL syndrome is a rare autosomal dominant syndrome characterized by multiple benign as well as malignant neoplasms. As discussed, mutations in the tumor suppressor gene *VHL*, located on chromosome 3 are pathognomic. It may present in childhood, during adolescence, or in adulthood, and is highly variable in its presentation, even within the same family (or those sharing the same pathogenic variant), with a mean age of initial presentation in the third decade.

The molecular pathogenesis follows the “two-hit” model. Affected individuals have a germline variant that causes inactivation of the *VHL* gene in all cells. The second “hit” is due to a somatic mutation or deletion of the second allele. Hypoxia-inducible factor 1 alpha (HIF1A) and 2 alpha (HIF2A) are two of the major proteins regulated by the *VHL* gene [5]. They are involved in angiogenesis, glucose transport, and erythropoiesis. Dysregulation of these proteins predisposes to tumor cell proliferation in multiple organs. It is also a tumor suppressor gene, and its inactivation leads to tumor proliferation. The various VHL-associated lesions and their relative incidence [4] are shown in Table 2.

**Table 2:** von Hippel-Lindau-associated tumors and the frequency with which they occur.

	Frequency
Retinal hemangioblastomas	25–60%
Endolymphatic sac tumors	10–25%
Cerebellar hemangioblastomas	44–72%
Brainstem hemangioblastomas	10–25%
Spinal cord hemangioblastomas	13–50%
Renal cell carcinoma or cysts	25–60%
Pheochromocytomas	10–20%
Pancreatic cysts/tumors	35–70%
Epididymal cystadenomas	25–60% of males

Based on the likelihood of developing pheochromocytoma [6], families with VHL syndrome are divided into the following two types: Type 1 - lower risk of developing a pheochromocytoma and renal cell carcinoma, although they have a higher risk of developing other associated lesions; and Type 2 - high risk for developing a pheochromocytoma.

The clinical features depend on the site and size of the tumors involved. Hemangioblastomas in the central nervous system (CNS), commonly occurring in the cerebellum and spinal cord can cause headaches, vomiting, sensory or motor deficits, ataxia, and other features of space-occupying lesions. Hemangioblastomas in the retina may cause vision loss. Pheochromocytomas or paragangliomas may be asymptomatic, but they may also cause a plethora of symptoms, including headaches, panic attacks, excessive sweating, and elevated blood pressure. VHL patients with endolymphatic sac tumors, occurring in the inner ear, may present with tinnitus, vertigo, or hearing loss.

Physical examination findings are usually limited as the diagnosis is largely based on laboratory and radiological studies. However, neurological findings such as muscle weakness, sensory deficits, and ataxia in the case of CNS hemangioblastomas may be present.

The diagnosis of VHL syndrome is typically made with the detection of a pathogenic variant of the *VHL* gene or a positive family history and one VHL-associated lesion [7]. If there is no family history and no access to genetic testing, the diagnosis may be made if two or more VHL-associated lesions are present. Therapy is mainly directed toward standard management of that specific tumor.

The optimal therapy for a pheochromocytoma is surgical resection of the tumor. However, it is associated with high rates of mortality if not properly prepared. Initial alpha-blocker treatment is crucial for controlling blood pressure in patients with pheochromocytoma. Once sufficient alpha-blockade is achieved, the patient can receive beta-blocker treatment to regulate heart rate. Phenoxybenzamine is the recommended alpha-blocker. Due to the release of catecholamines during intraoperative tumor handling and the effects of anesthetic medications, patients are susceptible to accelerated hypertension, hypotension, arrhythmias, and cardiovascular instability during the intraoperative phase.

Belzutifan, an HIF2a inhibitor, has recently been approved by the United States Food and Drug Administration [8] for use in VHL, especially renal cell carcinoma [9], CNS hemangioblastomas, or pancreatic neuroendocrine tumors, not requiring immediate surgery. The American Society of Clinical Oncology identifies VHL as a Group 1 disorder: an inherited disorder for which genetic testing is considered a part of the standard management for at-risk family members [10].

Our patient had a retinal hemangioblastoma, cystic lesions in his pancreas and both kidneys, and a pheochromocytoma. However, there was no family history of such lesions, other malignancies, or hypertension. Genetic testing was planned, but due to financial constraints, it has been deferred at present.

## Conclusions

The case discussed here is a case of VHL syndrome with a history of a retinoblastoma presenting with a pheochromocytoma, which is a rare combination. The presence of multiple cystic lesions in the pancreas and kidneys, especially in an individual with a family history of VHL, should alert the physician to the possibility of VHL syndrome. Classical lesions, especially hemangioblastomas of the cerebellum, retina, or spinal cord, should also raise suspicion of this syndrome. This case also underscores the importance of thorough evaluation for secondary hypertension, especially in younger patients. Clinicians should remain vigilant for unusual combinations of symptoms and imaging findings, which may indicate an underlying genetic disorder such as VHL syndrome. Early recognition and diagnosis are paramount for timely management and improved patient outcomes.
